# A proposal of good manufacturing practices (GMP) laboratory for CAR T-cell therapy in Latin America and the Caribbean: challenges and opportunities

**DOI:** 10.3389/fpubh.2026.1875553

**Published:** 2026-08-20

**Authors:** Sergio Camilo Cabrera-Salcedo, Luis Gabriel Parra-Lara, Monica Fernandes-Pineda, Alejandro Toro-Pedroza, Juan Sebastian Victoria Hincapie, Lady J. Rios-Serna, Alexandre Loukanov, Joshua Ortiz-Guzman, Juan Esteban Garcia-Robledo, Carlos A. Cañas, Gustavo Adolfo Cruz-Suarez, Felipe Ocampo Osorio, Michael P. Gustafson, Juan Guillermo Restrepo, Andres Mosquera, Valentina Hoyos, Juan C. Baena

**Affiliations:** 1 Facultad de Ciencias de la Salud, Universidad Icesi, Cali, Colombia; 2 LiliCAR-T Group, Fundación Valle del Lili, Icesi, Cali, Colombia; 3 Centro de Investigaciones Clínicas, Unidad de Inteligencia Artificial, Fundación Valle del Lili, Cali, Colombia; 4 Service of Clinical Oncology, Department of Internal Medicine, Fundación Valle del Lili, Cali, Colombia; 5 Universidad Icesi, CIRAT: Centro de Investigación en Reumatología, Autoinmunidad y Medicina Traslacional, Cali, Colombia; 6 Department of Chemistry and Materials Science, National Institute of Technology, Gunma College, Maebashi, Japan; 7 Laboratory of Engineering Nanobiotechnology, University of Mining and Geology "St. Ivan Rilski", Sofia, Bulgaria; 8 Minos LLC, Department of Research and Development, Miami, FL, United States; 9 Prodigy Cells Labs, LLC, Doral, FL, United States; 10 Centro de Investigación Hemato Oncológico (CIHO), IDC Instituto de Cáncer Hemato Oncólogos, Cali, Colombia; 11 Division of Rheumatology, Department of Medicine, Fundación Valle del Lili, Cali, Colombia; 12 Departamento de Salud Pública y Medicina Comunitaria, Universidad Icesi, Cali, Valle del Cauca, Colombia; 13 Department of Laboratory of Medicine and Pathology, Mayo Clinic in Arizona, Scottsdale, AZ, United States; 14 Center for Cell and Gene Therapy, Baylor College of Medicine, Texas Children's Hospital, and Houston Methodist Hospital, Houston, TX, United States

**Keywords:** artificial intelligence, CAR T-cell therapy, good manufacturing practices (GMP), health equity, Latin America and the Caribbean, modular laboratories, public health policy, regulatory harmonization

## Abstract

Chimeric antigen receptor (CAR) T-cell therapy is a transformative modality for refractory hematologic malignancies; however, its adoption in Latin America and the Caribbean (LAC) is severely constrained by prohibitive manufacturing costs, limited specialized infrastructure, and fragmented regulatory frameworks. To address this translational gap, we propose a pragmatic, cost-adapted model for establishing Good Manufacturing Practice (GMP) laboratories tailored to the socioeconomic realities of LAC. This model advocates for decentralized, modular cleanroom facilities that integrate fully closed automated manufacturing platforms with risk-based quality management systems. Furthermore, we emphasize the strategic implementation of artificial intelligence (AI) and digital health tools—such as electronic batch records and predictive analytics—to optimize resource utilization and ensure stringent regulatory compliance. Essential to this framework is the cultivation of a specialized local workforce and the promotion of regional regulatory harmonization through initiatives like PAHO’s Red PARF. Ultimately, this scalable approach provides a realistic roadmap to democratize CAR T-cell therapy in LAC, fostering regional biomanufacturing autonomy and promoting equitable patient access.

## 1 Background

**Figure 1:**
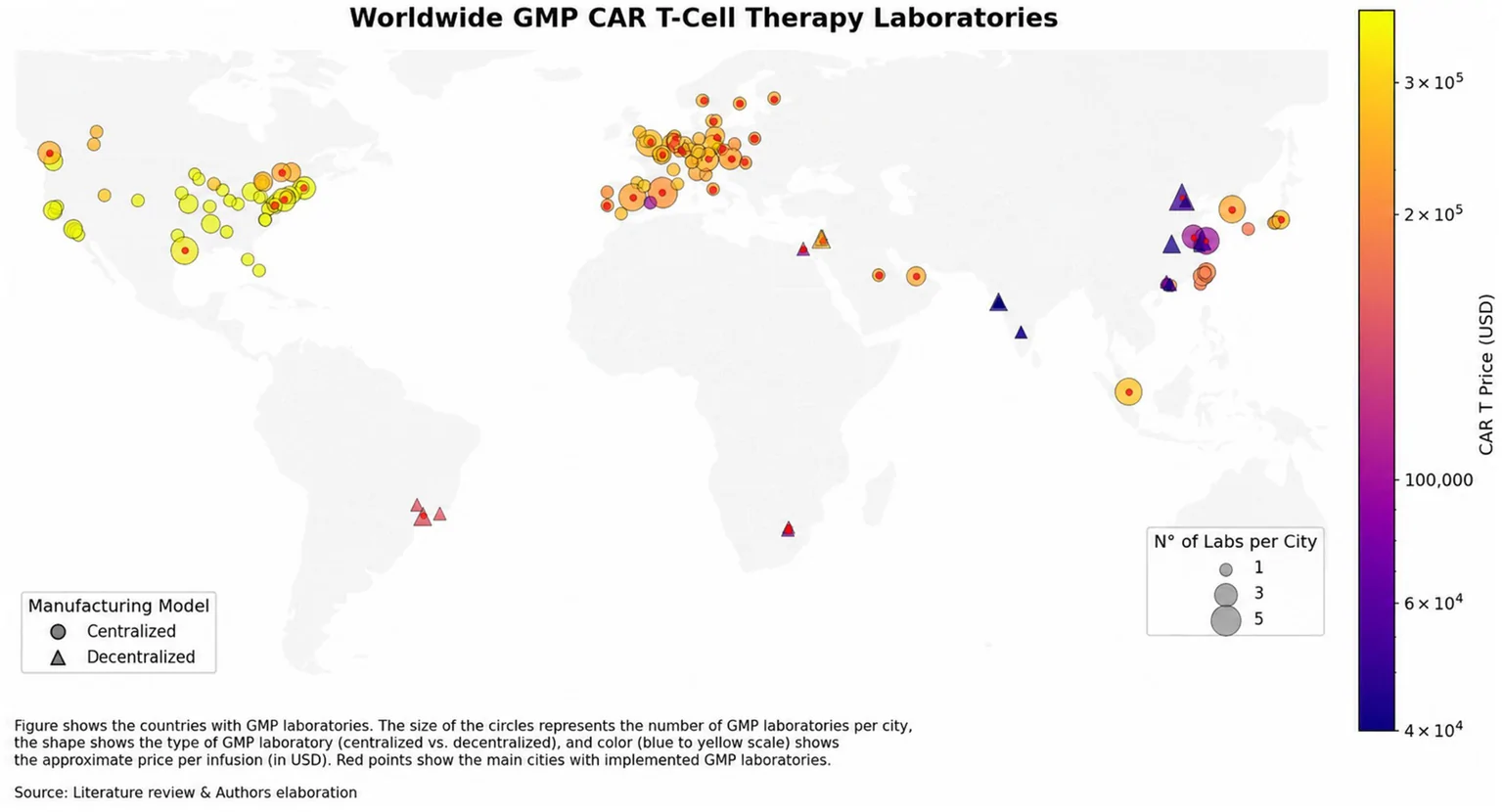
Countries with GMP CAR T-Cell Therapy Laboratories. The figure shows the countries with GMP laboratories, the size of the figures represents the number of GMP laboratories, the form shows the type of GMP laboratory (centralized vs. decentralized), and color (blue to yellow scale) the approximate price per infusion (in USD). Red points showed the main cities with implemented GMP laboratories. For additional details, see Supplementary Table 1. *This figure is illustrative and may not include all locations with GMP capacity; infusion prices are approximate and provided for comparative purposes only.

Good Manufacturing Practices (GMP) comprise the set of standards applied by entities producing drugs and biologic products, including cellular therapies, to ensure that these products consistently meet predefined requirements for identity, strength, quality, and purity (1). The WHO defines GMP as the component of quality assurance guaranteeing that medicinal products are manufactured and controlled to standards appropriate for their intended use (2). GMP was formally introduced by the FDA in 1962–1963, with the first regulations for finished pharmaceuticals (3). Decades later, the first clinical-grade CAR T-cell products were manufactured at the University of Pennsylvania in 2010 (4), and in 2017 Novartis received the first industrial-scale regulatory approval for a CAR T-cell therapy (5). To date, GMP laboratories remain concentrated predominantly in high-income countries, driven by greater access to funding, technological infrastructure, trained personnel, and mature regulatory frameworks. Latin America and the Caribbean (LAC), a region of 652 million inhabitants in 2023 (6), reports an estimated 1.5 million new cancer cases and approximately 700,000 deaths annually, with corresponding incidence and mortality rates of 186.5 and 86.6 per 100,000, respectively (7), yet faces substantial challenges in scaling GMP-compliant infrastructure despite its potential to develop local cell-therapy manufacturing capacity. A significant gap therefore remains between LAC and high-income countries in the implementation of GMP laboratories for CAR T-cell therapy (8, 9). Despite the growing clinical need, the regional distribution of GMP facilities capable of supporting CAR T-cell manufacturing remains highly uneven across LAC. Existing centers are concentrated in a limited number of countries, highlighting substantial disparities in infrastructure availability and reinforcing the need for regional expansion of GMP capacity (Figure 1). The objective of this review is to describe, analyze, and propose strategies for establishing GMP laboratories dedicated to CAR T-cell therapy in LAC, considering the region’s specific challenges, regulatory advances, and opportunities.

A GMP laboratory is a production and control facility designed, constructed, and operated to ensure that medicines, including advanced therapy medicinal products such as CAR T cells, are manufactured safely, reproducibly, and with certified quality (10). Cellular therapy manufacturing follows three main models. Centralized manufacturing relies on a limited number of specialized, high-capacity facilities that distribute products to treatment centers, enabling economies of scale but extending vein-to-vein time (10–15). Decentralized manufacturing distributes production across multiple, often hospital-based sites, shortening delivery times but introducing challenges in oversight and inter-site variability (1, 16–18). Hybrid models balance both approaches, requiring tightly coordinated chain-of-identity and chain-of-custody systems (13–15, 17). Process automation is similarly stratified: open systems require operator manipulation within Grade B/ISO 7 cleanrooms; semi-closed systems reduce, but do not eliminate, dependence on high-classification environments; and fully closed, automated systems integrate all steps into sealed, single-use workflows operable in Grade C/ISO 8 cleanrooms, minimizing contamination risk and potentially lowering costs (10, 14, 19, 20). Three principal installation types have been described: translational research laboratories suited to Phase I/II trials; commercial production plants operating at high capacity under full GMP certification; and modular or mobile cleanroom platforms, which offer shorter construction timelines and easier scalability, of particular value in infrastructure-limited settings (1, 21, 22). Facilities are further differentiated by process-flow specialization (integrated vs. segmented) and by governance model, with public facilities typically supporting investigator-initiated trials and private facilities, operating as CMOs, offering scalability at higher cost (11, 23–25). An integrated overview of the principal facility configurations, infrastructure components, operational workflow, and equipment required for GMP-compliant CAR T-cell manufacturing is provided in Supplementary Figure S1.

Establishing a GMP facility requires cleanrooms engineered to limit particulate and microbial contamination per ISO 14644 standards, classified into Grades A–D under the EMA system (Supplementary Table S2), with Grade A/ISO 5 reserved for the highest-risk aseptic operations and Grade D/ISO 8 for general production areas (10, 26–34). Architectural design must promote unidirectional personnel and material flow, progressive gowning, and strict environmental controls, including HEPA filtration (removing at least 99.97% of particles of 0.3 μm or larger), positive pressure cascades of 10–15 Pa between zones, and defined temperature (15–25 °C) and humidity (30–60% RH) ranges (Supplementary Figure S2) (20, 22, 35–41). Critical equipment, including biosafety cabinets, CO₂ incubators, magnetic cell-separation systems, closed expansion platforms, and controlled-rate freezers (Supplementary Table S3), together with qualified consumables and reagents, must undergo documented qualification, comprising Installation, Operational, and Performance Qualification, and continuous environmental monitoring to safeguard product sterility, potency, and traceability (10, 20, 42).

Quality control (QC) is the process ensuring that products meet predefined quality standards, defined by the WHO as the sum of all procedures undertaken to verify a product’s identity and purity (43). Per FACT–JACIE recommendations, QC must be integrated into every stage of manufacturing, from incoming material qualification to final release, encompassing sterility testing, mycoplasma detection, replication-competent virus assays, endotoxin quantification, immunophenotyping, and viability assessment (Supplementary Figure S3) (1). EudraLex Volume 4, Annex 1, together with WHO Technical Report Series 961, further emphasize continuous environmental monitoring and predefined alert and action limits, given that CAR T-cell products cannot undergo terminal sterilization (10, 20). QC units must operate independently from production, with authority to reject any non-conforming batch, an especially critical safeguard given the autologous, irreplaceable nature of each CAR T-cell product. A parallel traceability system, mandated by FACT–JACIE, European, and WHO guidance, ensures verifiable chain-of-identity and chain-of-custody records across the entire product lifecycle, from donor cell collection through manufacturing, quality control, storage, transport, and administration, enabling effective recall, root-cause investigation, and pharmacovigilance when needed (1, 10, 20).

GMP facilities require a multidisciplinary personnel structure, including a Processing Facility Director, holding a medical, doctoral, or equivalent degree, responsible for SOPs and quality management compliance; a Facility Medical Director overseeing clinical aspects; an independent Quality Manager; and adequately staffed, trained operators with designated backup coverage, as detailed under FACT–JACIE Standards (8th edition, v8.3) (1). This international standard, maintained since 1998 by the Joint Accreditation Committee ISCT–EBMT (JACIE), applies to all stages of the cellular therapy workflow across standard-of-care, clinical-trial, and licensed products (1, 18). At the national level, the FDA oversees Current Good Manufacturing Practice (CGMP) compliance in the United States, while the EMA coordinates GMP inspections and harmonization across the European Union; facility-level accreditation is further provided by specialized bodies such as FACT, CAP, and AABB (27, 28). Internationally, GMP standards are promoted by the WHO, while PAHO facilitates regional harmonization in the Americas through the Red Panamericana para la Armonización de la Reglamentación Farmacéutica (Red PARF), which strengthens member countries’ regulatory systems and builds regulatory science competencies (44). The principal international regulatory and accreditation frameworks governing GMP-compliant CAR T-cell manufacturing are summarized in Supplementary Figure S4.

Taken together, these internationally established standards define a demanding, resource-intensive template for CAR T-cell manufacturing that has, to date, been implemented almost exclusively within high-income health systems. The remainder of this review addresses the central question that these requirements raise for LAC: how can a region characterized by pronounced economic disparity, regulatory heterogeneity across 33 national jurisdictions, and constrained biomanufacturing infrastructure realistically adapt this template rather than simply replicate it? The following sections examine the strategic rationale for AI-enabled manufacturing in resource-constrained settings, and then develop a phased, modular, and cost-adapted implementation model, addressing facility design, automation-platform selection, human-capital development, intellectual-property barriers, and viral-vector supply, culminating in the regional roadmap that constitutes the central contribution of this work.

## 2 Strategic context and regional rationale for AI-enabled CAR-T manufacturing in Latin America

Artificial intelligence (AI) constitutes a pragmatic enabler for CAR-T cell manufacturing in resource-constrained environments across LAC, where limitations in patient-derived starting material, specialized personnel, and cleanroom availability restrict scalability. In this setting, the primary value of AI lies in improving prediction, process control, and documentation rather than in full automation. Effective implementation therefore requires fit-for-purpose digital architectures aligned with regional regulatory frameworks (ANVISA, INVIMA, COFEPRIS) and adaptable to heterogeneous institutional capabilities (45).

A staged adoption pathway beginning with structured data capture, descriptive analytics, and electronic batch records (eBRs), and progressively advancing toward real-time process analytical technologies (PAT), soft sensors, and digital twins offers a technically feasible and economically sustainable strategy for LAC providers. This incremental model aligns with risk-based quality management principles such as ICH Q9(R1) and enables early identification of manufacturing deviations and supply-chain vulnerabilities across diverse healthcare systems, from academic GMP units to public-sector facilities.

AI delivers particular benefit in upstream operations unique to autologous CAR-T workflows. Predictive models integrating pre-apheresis clinical and hematologic variables can estimate CD3^+^ yields and improve the probability of meeting manufacturing thresholds, reducing failed runs and inefficient resource utilization (46). In parallel, image-based machine-learning approaches linking immunological synapse architecture to clinical outcomes suggest that AI-assisted potency assessment may shorten release timelines once appropriately validated under GMP conditions (47). Beyond process optimization, AI-driven computational immunology—including integrative immunopeptidomic and proteogenomic frameworks—supports systematic identification of tumor-associated antigens presented by HLA molecules, expanding the actionable antigen repertoire while improving specificity and safety in personalized CAR-T design. These applications are particularly suited to public–private collaborations in LAC, enabling localized data generation while fostering regional computational expertise (48).

Among the most immediately impactful applications of AI in CAR-T manufacturing is the digitalization of quality systems. Validated eBRs, digital audit trails, and exception-flagging algorithms enhance quality assurance efficiency in facilities with limited QA staffing, while supporting compliance with 21 CFR Part 11, EU GMP Annex 11, and regional regulatory equivalents. Standardized eBR templates and risk-based computerized system validation may further reduce regulatory burden and inspection rework for LAC agencies. AI-enabled quality control integrates multivariate process data to detect deviations in cell growth, viability, reagent performance, and chain-of-identity integrity prior to product release—an essential safeguard in autologous therapies, where each manufactured lot corresponds to a single patient and is inherently non-replaceable. Integration of manufacturing and clinical datasets further enables patient stratification and prioritization of manufacturing resources, an important advantage in capacity-limited settings.

However, uneven AI adoption risks exacerbating existing disparities in access to CAR-T therapies across LAC, where institutional digital maturity varies widely. Algorithmic bias may arise from overrepresentation of data from urban private centers, class imbalance in outcome datasets, and incomplete or lower-quality data from under-resourced institutions, limiting generalizability and fairness. Consequently, data quality, diversity, and governance are as critical as model performance. Responsible AI deployment requires multicenter validation across heterogeneous populations, explicit fairness metrics, routine algorithmic audits, and multidisciplinary ethical oversight. Inclusion of sociodemographic variables may improve personalized risk stratification but must be carefully governed to avoid reinforcing structural inequities. In this regard, PAHO’s digital health and AI principles provide a regional policy scaffold for equitable, interoperable, and privacy-preserving implementation (49, 50).

Effective deployment of AI-enabled analytics in LAC CAR-T programs rests on three foundational requirements: adequate computational infrastructure to process large clinical, manufacturing (51), and multi-omic datasets through secure cloud or high-performance computing environments (52); robust data governance frameworks ensuring quality, traceability, consent, and oversight of automated decision-making (53–57); and interoperability standards that enable integration of heterogeneous data streams through shared data dictionaries and harmonized terminologies (e.g., SNOMED CT, LOINC). Absent these foundations, advanced analytics risk opacity, inefficiency, and safety failures.

Finally, the convergence of AI with nanotechnology and microfluidics offers a realistic pathway toward intelligent GMP laboratories adapted to resource-limited environments (58–60). Nanosensors integrated into bioreactors enable continuous, non-invasive monitoring of metabolic and immunological parameters (61–63), while lab-on-a-chip platforms miniaturize functional assays and reduce reagent and personnel requirements (64, 65). AI amplifies the value of these systems by enabling real-time anomaly detection, prediction of critical quality attributes, and closed-loop optimization of culture conditions under GMP constraints (66, 67). As data maturity increases, hybrid digital-twin models combining mechanistic understanding with data-driven inference provide a scalable route toward model-predictive control of CAR-T expansion processes, compatible with single-use and hospital-embedded manufacturing systems common in LAC (68, 69).

## 3 Strategic proposal for the implementation of GMP laboratories for CAR t-cell therapy in Latin America and the Caribbean

LAC comprises 33 countries, each governed by distinct regulatory and normative authorities. This pronounced regulatory heterogeneity represents a major structural challenge for the implementation of CAR T-cell therapy, as countries differ substantially in the availability of regulatory frameworks, technical expertise, and standards for quality, safety, and efficacy. A comparative overview of regulatory pathways in Argentina, Brazil, Colombia, and Mexico is presented in Table 1, highlighting that, in all cases, laboratories require explicit national authorization to operate in addition to compliance with international GMP standards. Beyond regulatory compliance, the physical infrastructure of manufacturing facilities must also meet specific requirements to support GMP-compliant operations. In resource-limited settings, where facility design is often constrained by budget, space, or construction standards, establishing minimum functional dimensions for each production area becomes a critical planning parameter. Standardized spatial references can guide institutions and policymakers in designing facilities that are both regulatorily compliant and operationally viable within the regional context. Proposed laboratory dimensions adapted to the regional context are summarized in Table 2.

**Table 1:** Comparison of regulatory normative in 4 countries of Latin America and the Caribbean.

Country	Regulatory entity	Regulatory normative in GMP	Approach and scope
Argentina	ANMAT	Provision 4159/2023 Resolution 610/2007	GMP mandatory for injectable products Supervises products with cellular therapeutic potential INCUCAI participates in cases of cell transplantation
Brazil	ANVISA	RDC 9/2011 RDC 56/2010	Strictly regulates Cell Technology Centers (CTCs) for therapy and clinical trials GMP adapted to cellular processing activities
Colombia	INVIMA	Resolution 1160/2016	Adopts WHO guidelines Requires GMP certification for laboratories Covers therapies under the oversight of cellular biological products
Mexico	COFPRIS / CNTS	Normas derivadas de NOM-059-SSA1-2015 (medicamentos estériles) Regulaciones CNTS para CPH y terapias avanzadas	Annual regulatory evaluation and monitoring by CNTS GMP compliance required for establishments with COH Control and certification through COFEPRIS

INCUCAI, Instituto Nacional Central Único Coordinador de Ablación e Implante impulsa.

**Table 2:** Proposed dimensions for a GMP laboratory in LAC.

Area	Space (m^2^)
Changing rooms/gowning/access/transition corridors	40–70 m^2^
Primary production room (ISO-7 with ISO-5 cabinets/hood)	70–100 m^2^: 3 modules of 22–30 m^2^ (each module → sterile work area with BSC and equipment)
Preparation and testing area (Grade C/D)	40–60 m^2^
Analytical QC (qPCR, cytometry, endotoxins, micro)	60–100 m^2^
Raw material warehouse (controlled) + CRT chamber + LN₂ cryostorage	50–90 m^2^
Formulation/filling and shipping room (ISO-5/ISO-7)	25–40 m^2^
Auxiliary services (HVAC technical room, UPS, QA office, meeting)	70–150 m^2^

CRT, climate room test; HVAC, heating, ventilation, and air conditioning; ISO, international organization for standardization; LN_2_, liquid nitrogen; QA, quality assurance; QC, quality control; qPCR, quantitative polymerase chain reaction; UPS, uninterruptible power supply.

Beyond regulatory complexity, the region is characterized by marked economic disparities and heterogeneous healthcare systems, which further constrain the deployment of advanced cell therapies. Even when access to automated GMP cell-processing platforms is available—such as the CliniMACS Prodigy® TS 520, a sterile, flexible, reproducible, closed, and configurable system with built-in safety and traceability—overall costs remain prohibitively high. These financial barriers, compounded by logistical challenges related to supply chains, infrastructure, and workforce availability, significantly limit the widespread adoption of CAR T-cell therapy in LAC (70).

Recent advances in automation have nevertheless created opportunities to improve reproducibility and partially mitigate costs. First-generation platforms enhanced efficiency through robotic arms and automated liquid handling, facilitating scale-out manufacturing. More advanced second-generation systems integrate multiple manufacturing steps into fully closed platforms, enabling continuous monitoring, reducing contamination risk and operator-dependent variability, and potentially lowering infrastructure costs by decreasing reliance on high-grade cleanrooms. Their modular and flexible design is particularly well suited to the diverse and evolving landscape of cell and gene therapy manufacturing in LAC (71), as summarized in Table 3.

**Table 3:** Comparative overview of first- and second-generation automated platforms for cell and gene therapy manufacturing.

System/platform	Generation	Main features	Advantages	Limitations
Freedom EVO® (Tecan Group Ltd., Männedorf, Switzerland)	1st	Pipetting robot, liquid handling	High precision, faster processing, liquid detection	Requires specific consumables, needs special programming training
STAR (Hamilton Reno, Nevada, USA)	1st	Modular pipetting platform	High precision for small volumes, expandable design	Handles only ≤5 mL at a time, requires specific consumables
CompacT SelecT™ (Sartorius, Göttingen, Germany)	1st	Incubator + robotic arm + pumps; adherent & suspension culture	Processes up to 90 flasks; subculture, counting & harvesting	Needs centrifuge/microscope externally, large footprint
Biomek 4,000 (Beckman Coulter Inc., Brea, California, USA)	1st	Automated pipetting workstation	High accuracy for small volumes	Requires specific consumables
RoboLector (M2P Labs, Baesweiler, Germany)	1st	Automated cultivation in microplates, pH adjustments	High-throughput, integrated analytics	Limited to small volumes; not validated for human cells
Cellmate (Sartorius/TAP Biosystems, Royston, United Kingdom)	1st	Expansion in flasks/roller bottles	Suitable for higher volume expansion	No automated harvesting
CyBio (Analytik Jena, Jena, Germany)	1st	Full assay automation	Automates seeding, incubation & plate assays	Only microplates, specific consumables
Ambr® 15 & Ambr® 250 (Sartorius, Göttingen, Germany)	1st	Disposable bioreactor vessels; scale-down models	High throughput, multiple conditions simultaneously	Limited agitation speeds, specific consumables
CliniMACS Prodigy® (Miltenyi Biotec, Bergisch Gladbach, Germany)	2nd	Fully closed, modular; autologous cell therapy	Activation, transduction, expansion, harvest in one unit; EMA approved for CAR-T/Zalmoxis	Only suspension cells; limited to small volumes; bottleneck in long expansion
Cocoon ™v (Octane Biotech Inc., Lucknow, India)	2nd	Closed, all-in-one chamber; CAR-T focus	Automated seeding, expansion, perfusion, harvest, formulation	Tested only for CAR-T; no adherent culture
Quantum (Terumo BCT Inc., Lakewood, Colorado, USA)	2nd	Hollow-fiber bioreactor (2.1 m^2^ cartridge)	Tested with MSCs, ASCs, neural stem cells	Only expansion step; no enrichment or isolation
Stem Cell Factory (Fraunhofer Project Center for Stem Cell Process Engineering, Würzburg, Germany)	2nd	Automated iPSC production	Can process up to 60 lines in parallel	Limited to iPSC reprogramming/differentiation
AUTOSTEM Platform	2nd	Fully enclosed, robotic arms, modular grippers	Donor-to-patient workflow: collection, isolation, expansion, QC, cryopreservation; adaptable to adherent & suspension cells	Still under development, complex integration

ASC, Adipose-derived Stem Cells; CAR, Chimeric antigen receptor; EMA, European Medicines Agency; iPSC, induced pluripotent stem cells; MSC, Mesenchymal Stem Cells; QC, quality control.

Country-level experiences further illustrate these challenges and opportunities. Colombia, for example, with a population of approximately 53 million and around 14 HSCT centers, reflects the broader regional situation. Establishing a GMP facility requires careful evaluation of process-change costs, workforce demands, and the availability of highly skilled personnel. While CAR T-cell administration is optimally performed within HSCT programs to ensure clinical readiness, GMP manufacturing facilities do not necessarily need to be physically collocated. Instead, the development of integrated manufacturing hubs in selected LAC countries, with subsequent distribution to affiliated HSCT centers, may represent a more efficient and cost-effective regional strategy (72).

Emerging initiatives in Brazil provide a compelling proof-of-concept for localized CAR T-cell manufacturing in LAC, transitioning decentralized models from theory to reality. In 2024, the Oswaldo Cruz Foundation (Fiocruz) established a strategic public-private partnership with the non-profit Caring Cross to develop local manufacturing capacity for CAR-T cells and lentiviral vectors at the Bio-Manguinhos facility (73). By leveraging a point-of-care production model within the public health system, this initiative projects a cost reduction of approximately 90% compared to commercial US and EU therapies (74). Similarly, the Nutera Advanced Therapy Center—a collaboration between the Butantan Institute and the USP Ribeirão Preto Blood Center—opened in 2022 as Latin America’s first dedicated ATMP plant. Supported by ANVISA, this facility has already initiated Phase I/II clinical trials for CAR-T therapies targeting acute lymphoblastic leukemia and non-Hodgkin lymphoma, acting as a foundational supplier for Brazil’s Unified Health System (SUS). These programs underscore the viability of combining centralized regulatory oversight with hospital-embedded processing to dramatically reduce costs and expand access (75).

Affordability remains the most critical barrier to CAR T-cell implementation in LAC, yet comparatively lower clinical trial costs and judicious investments in facility design, automation, and regional coordination can reduce development expenditures. In this regard, the integration of AI into GMP laboratory operations is a promising enabler: AI can support real-time monitoring of bioreactor conditions, predictive modeling of cell expansion and transduction efficiency, early detection of process deviations, and automated, regulatory-compliant documentation, all of which may improve reproducibility and accelerate product release. However, AI should be understood as a multiplier of efficiency rather than a substitute for foundational infrastructure and human capital. Put differently, the benefits of AI accrue only insofar as they are embedded within operational models that reflect local regulatory realities, constrained budgets, supply-chain fragilities, and workforce availability.

These structural, economic, and technological realities therefore argue against simple replication of high-income country GMP templates in LAC. Instead, they point to the need for a phased, adaptive model—one that leverages automation and AI to maximize efficiency where feasible, while relying on modular facility design, regional hubs for specialized assays, and public–private partnerships to distribute costs and concentrate technical expertise. Collectively, these considerations underscore the imperative for a tailored, region-specific model for GMP laboratories capable of supporting scalable, sustainable CAR T-cell therapy in LAC.

**Figure 2:**
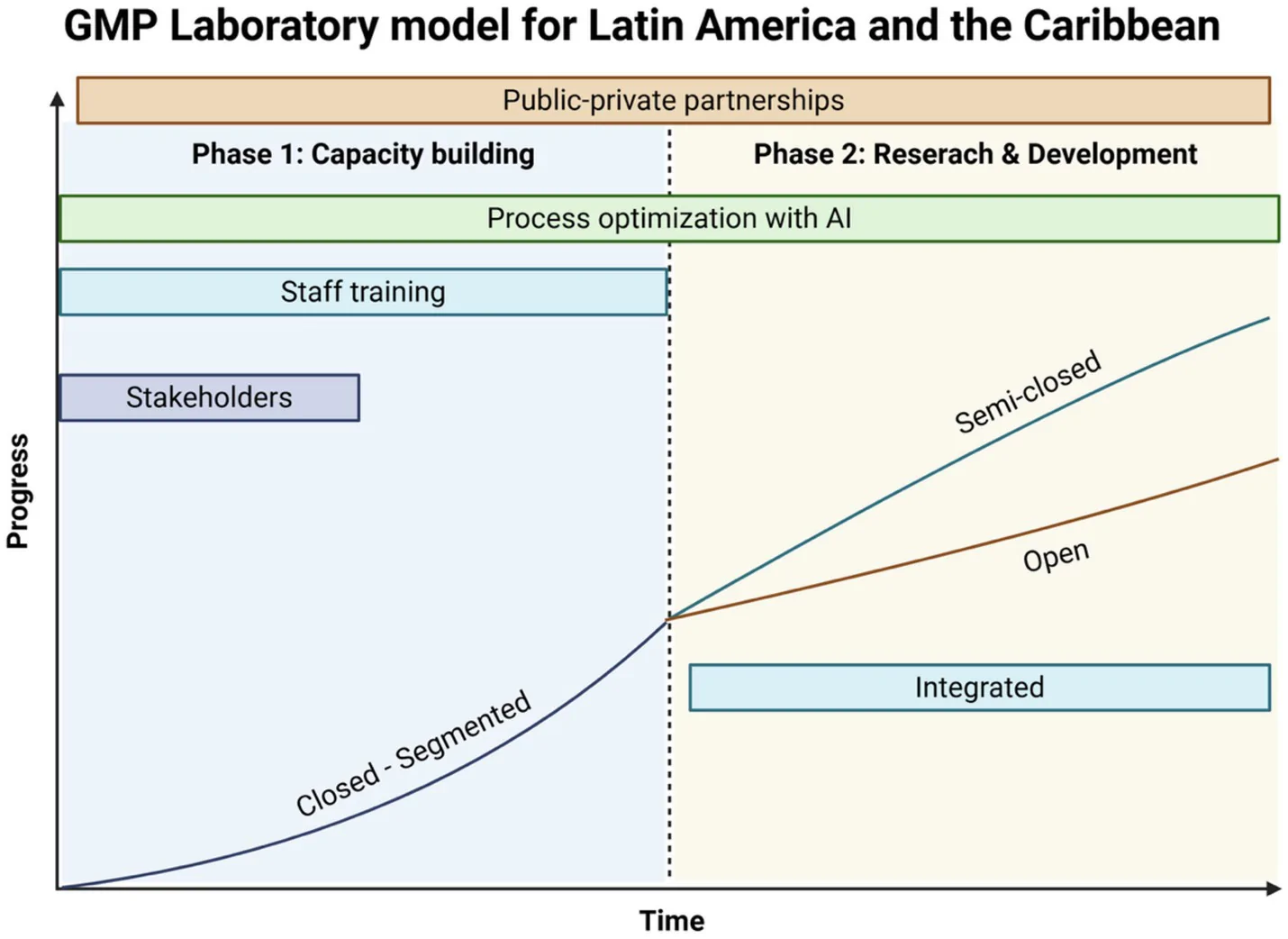
Implementation model for a GMP laboratory for LAC. The proposed implementation model follows a two-phase strategic roadmap. Phase 1 focuses on capacity building through a segmented, closed-system GMP laboratory design, prioritizing staff training and basic operational readiness. Phase 2 transitions toward a hybrid model (integrating both open and semi-closed systems) with fully consolidated processes aimed at advanced Research & Development. Throughout both phases, Artificial Intelligence (AI) serves as a transversal enabler to continuously optimize laboratory workflows. The financial sustainability of the facility is envisioned to be supported through robust public-private partnerships.

The establishment of GMP laboratories for CAR T-cell therapy in LAC requires a model that balances technical rigor, economic sustainability, and regional equity. Given the heterogeneity of infrastructure, fragmented regulatory frameworks, and limited financial capacity, a direct replication of high-income country facilities is impractical. Instead, a phased and adaptive strategy is recommended. Figure 2 illustrates a proposed implementation framework adapted to the regional context.

**Figure 3:**
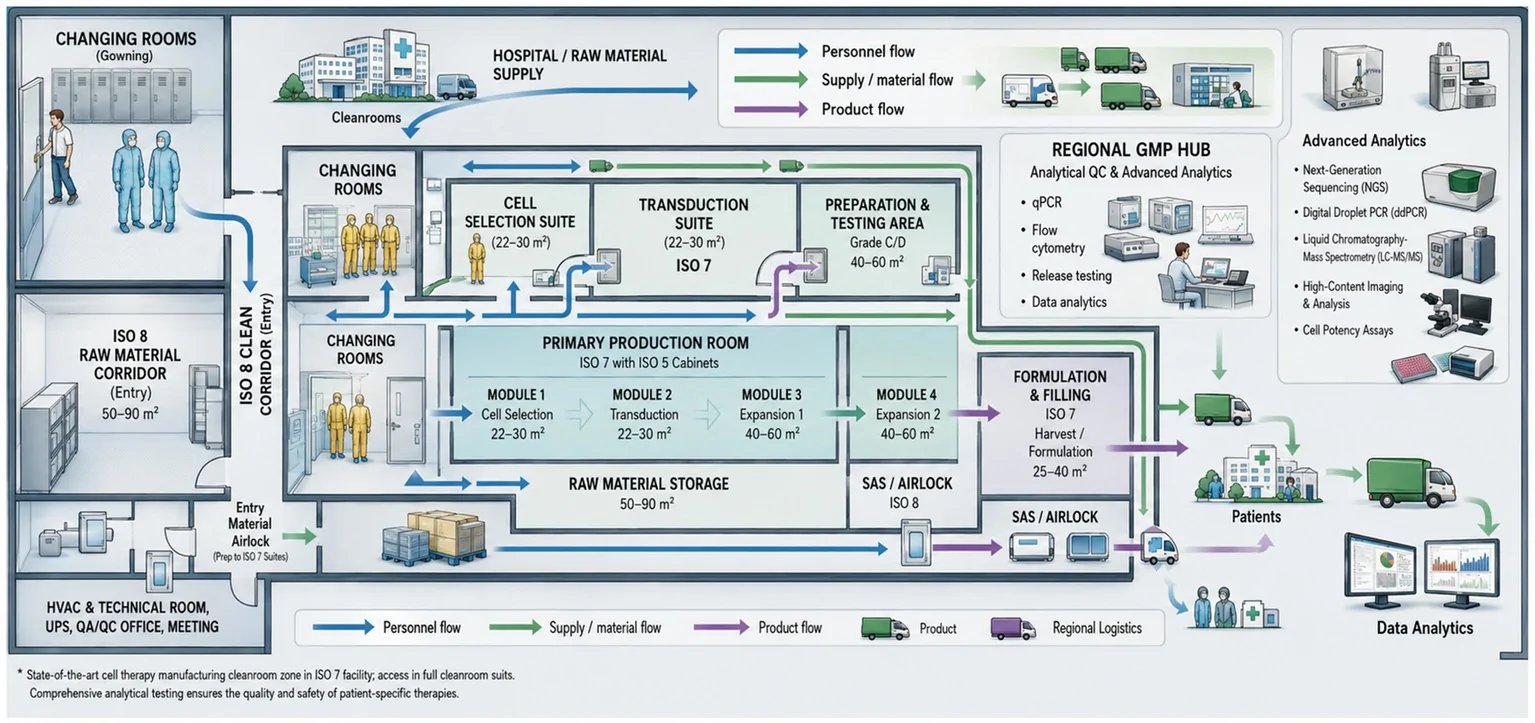
Spatial distribution and workflow of a GMP facility for cell therapy manufacturing. The design follows a linear sequence: entry through gowning and preparation areas, progression to ISO-7 production rooms with ISO-5 biosafety cabinets, expansion modules, and formulation/filling, followed by controlled freezing, cryostorage, quality control, and final product release for shipping. HVAC: Heating, ventilation, and air conditioning; GMP: Good manufacturing practice; ISO: International Organization for Standardization; qPCR: Quantitative polymerase chain reaction; UPS: Uninterruptible power supply.

A phased model should initially rely on a segmented structure, outsourcing vector production and highly specialized assays while performing critical steps such as leukapheresis, T-cell activation, transduction, expansion, formulation, and cryopreservation locally. Over time, this structure can transition toward fully integrated systems, fostering regional autonomy, stimulating biotechnology entrepreneurship, and driving innovation. Hybrid facilities combining modular, rapidly deployable interiors with more permanent external structures provide a cost-effective and scalable solution. A footprint of 400–700 m^2^ is sufficient to accommodate three ISO-7 suites equipped with ISO-5 biosafety cabinets, together with designated areas for formulation, cryopreservation, and QC. QC capabilities should initially include flow cytometry, PCR/ddPCR assays, sterility and endotoxin testing, while more advanced analytics—such as next-generation sequencing (NGS)-based integration site analysis—could be centralized in regional hubs. Figure 3 shows a proposed spatial distribution for a CAR T-cell manufacturing facility, including personnel flow, material flow, and ISO-classified cleanroom areas.

Automation is another cornerstone for implementation in LAC. Closed platforms such as CliniMACS Prodigy® and Lonza Cocoon® offer important advantages, including minimized contamination risk, reduced cleanroom classification requirements, and faster regulatory approval. However, dependence on proprietary consumables can increase costs and reduce autonomy. Conversely, open or semi-open processes provide greater flexibility but require more complex infrastructure and highly trained staff. A hybrid approach, employing closed systems for early clinical access and small open research modules for innovation, appears to be the most feasible pathway for the region.

Regarding scalability, both platforms present limits inherent to their single-unit (“black-box”) design. The Prodigy operates with a 250 mL culture chamber and a maximum capacity of 5 × 10^9^ total cells, with a smaller target-cell input capacity (3 × 10^9^) than the CliniMACS Plus (10 × 10^9^), and is less scalable than platforms such as the Xuri or G-Rex, which are available with larger culture volumes (76). The Cocoon Platform, using a single-use cassette, achieves an average 104-fold expansion (range 82–142) and a yield of approximately 2.3 × 10^9^ cells over a 10-day cycle; because each unit processes one patient batch at a time, scaling production requires proportional multiplication of identical units, translating scalability directly into linear capital investment rather than process efficiency (77). Selecting either platform also entails structural dependence on the manufacturer’s distribution chain, technical support, maintenance, and spare parts: reagents and tubing/cassette kits are proprietary consumables designed exclusively for each machine, and troubleshooting, critical in processes that run continuously over several days, requires close coordination with the equipment vendor, which can generate costly production delays and, at times, raise intellectual-property constraints. This single-sourcing risk has been widely documented in cell and gene therapy manufacturing, as these platforms depend on highly specialized supply chains, often from a single vendor offering customized technology produced by a small number of manufacturers. Notably, in April 2026, Lonza divested the Cocoon® Platform back to Octane Medical Group, the platform’s original developer, as part of Lonza’s transition to a pure-play CDMO business model; the platform therefore remains commercially available and supported, now under Octane Medical Group rather than Lonza, and LAC institutions considering this technology should direct procurement and support inquiries accordingly.

Economically, the decision between purchasing a closed commercial system, developing in-house capacity, or hiring/training specialized personnel is non-trivial. Automation lowers personnel costs, cleanroom classification requirements, and spatial footprint; automated Cocoon-based manufacturing, for instance, showed labor costs of roughly 60% of an equivalent manual process (77). However, cost modeling (Biosolve-based) indicates that while a partially automated process is the most economical configuration by cost-of-goods (CoGs) at smaller scale, the manual process can become more cost-effective once scaled to meet a larger annual demand (e.g., 5,000 patients) when the time-value of production is incorporated through net-present-cost evaluation, the same analysis recommends a “make-or-buy” sensitivity assessment for critical inputs such as viral vector and QC testing. Recruitment and retention of skilled manufacturing personnel remains one of the main obstacles for academic CAR-T manufacturers, who compete for scarce talent against industry; on-the-job training and university partnerships can help close this gap, offering an alternative to hiring already-trained staff or sending in-house personnel abroad for vendor-led training. A particularly cost-effective intermediate strategy for resource-limited settings such as LAC is to configure a hybrid, semi-open architecture: using the CliniMACS Prodigy only for the highest contamination-risk steps (cell selection and transduction), combined with lower-capital-cost bioreactors, such as the G-Rex, for the expansion phase, reducing overall dependence on a single vendor without forfeiting the benefits of automation at the most critical steps. Initiatives such as the European AIDPATH project illustrate this trend, integrating a scalable bioreactor (Aglaris Facer) with AI-driven, end-to-end automation modules within a “smart manufacturing hospital” model, rather than relying on a single closed commercial platform (78). This integration of process equipment, sensors, actuators, real-time data analytics, and control algorithms is precisely the core of Process Systems Engineering (PSE) applied to precision medicine, an approach identified as key to reducing the cost and time of autologous CAR-T manufacturing through smart manufacturing (Industry 4.0) (79).

Human resources are a critical determinant of sustainability. Experiences from hospital-based programs in Europe and the United States demonstrate that compact teams of 8–10 highly trained professionals can successfully operate small-scale GMP facilities treating 20–30 patients annually (80, 81). A proposed model for LAC would include a manufacturing lead responsible for production oversight, GMP compliance, and training; two to three GMP operators cross-trained in aseptic manipulation and automated platforms; a QA/QC lead overseeing documentation and product release; one to two QC analysts performing sterility, endotoxin, PCR, and flow cytometry assays; a clinical-logistics coordinator managing apheresis, cryoshipping, and chain-of-custody; and one to two data or bioinformatics specialists responsible for electronic batch records, AI-driven quality control, and predictive analytics. Such a model balances operational efficiency and regulatory compliance while enabling academic integration through the inclusion of postgraduate trainees. This staffing model balances operational efficiency with regulatory compliance while allowing the incorporation of postgraduate students, residents, and fellows, thereby strengthening academic training and capacity building in biotechnology.

Public–private partnerships (PPPs) will be essential. Universities and tertiary hospitals can provide infrastructure and clinical expertise, while industry partners contribute automation platforms, financing, and regulatory support. Regional harmonization initiatives such as PAHO’s Red PARF should be leveraged to streamline regulatory processes and reduce redundancy across agencies including INVIMA (Colombia), ANMAT (Argentina), ANVISA (Brazil), and COFEPRIS (Mexico). Establishing regional GMP hubs in select cities with existing technical capacity would further facilitate a phased and scalable implementation process.

Intellectual property (IP) constitutes one of the most significant structural barriers to CAR T-cell manufacturing in LAC, yet it is rarely addressed explicitly in implementation proposals. The core components of clinically validated CAR constructs—including scFv binding domains, co-stimulatory elements such as 4-1BB and CD28, and intracellular signaling regions—are covered by extensive patent portfolios held by originator companies including Novartis, Kite/Gilead, and Juno/Bristol Myers Squibb (82). Even in jurisdictions such as Brazil, where legislation excludes living organisms per se from patentability, companies routinely secure protection over formulations and manufacturing methods that effectively encompass these constructs. Consequently, academic or public-sector institutions in LAC cannot legally produce approved CAR T-cell designs without licensing agreements, which are often prohibitively expensive or simply unavailable to resource-limited settings.

Several complementary strategies may allow LAC institutions to navigate this landscape. The most sustainable long-term approach is the in-house development of novel, non-infringing CAR constructs. Academic groups such as those at the Ribeirão Preto Center for Cell Therapy and the Butantan Institute in Brazil are already engineering second- and third-generation CARs that circumvent originator patents while simultaneously building regional biotechnology expertise. A second avenue involves hospital exemption and compassionate use pathways. In the European Union, non-commercial CAR T-cell therapies can be supplied under special regulatory frameworks that bypass full market authorization; the Spanish ARI-0001 product, developed at Hospital Clínic de Barcelona and approved via this pathway, was made available at approximately one-third the cost of commercial alternatives (81). Non-profit and access-oriented licensing models offer additional precedent: the Caring Cross-Fiocruz partnership explicitly grants technology transfer and materials to Brazil’s public sector for CAR-T production, demonstrating operational feasibility in resource-limited settings (74). Regional licensing consortia, where LAC institutions or governments jointly negotiate patent rights, could distribute costs and strengthen bargaining power, following precedents in other high-cost therapeutic areas.

Viral vector supply presents an equally critical challenge. Clinical-grade lentiviral vectors—the primary gene delivery vehicle for CAR-T manufacturing—are produced at GMP level by a limited number of specialized CDMOs worldwide, including Oxford BioMedica, Miltenyi Biotec (formerly Lentigen), Waisman Biomanufacturing, and Cobra Biologics. This concentration creates supply-chain vulnerability and cost barriers for LAC programs. Short-term strategies should prioritize multi-institutional or government-negotiated supply agreements with existing CDMOs. Long-term regional autonomy, however, requires domestic GMP-grade vector manufacturing capacity. Brazil has initiated this transition: Bio-Manguinhos (Fiocruz) is establishing in-house lentiviral vector production under its Caring Cross partnership (83), and the Nutera Advanced Therapy Center (Butantan/USP) has built a platform supporting both viral vector and CAR-T manufacturing (84). A coordinated regional strategy, such as multi-country bioproduction hubs sharing capital investment, could ensure sustainable supply while preventing duplication. Failure to address intellectual property and vector supply proactively risks rendering even well-equipped GMP facilities unable to produce clinically actionable CAR-T products.

Finally, AI should be integrated as a transversal enabler across manufacturing and regulatory operations. AI applications include real-time monitoring of bioreactor conditions, predictive modeling of cell expansion and transduction efficiency, early deviation detection, automated GMP-compliant documentation (21 CFR Part 11manufactur), and optimization of logistics and scheduling (85–88). By reducing variability, accelerating release testing, and lowering costs, AI has the potential to expand access to CAR T-cell therapy and transform the feasibility of advanced therapies in LAC.

## 4 Conclusion and future directions

The establishment of GMP laboratories for CAR T-cell therapy in LAC represents both an urgent need and a unique opportunity. The current gaps in infrastructure, regulatory harmonization, and financial sustainability have limited access to these transformative therapies across the region. Our analysis indicates that implementing segmented, modular, and hybrid GMP facilities—supported by public–private partnerships and regional regulatory convergence—could significantly reduce costs while maintaining international quality standards. Importantly, the integration of automated and closed systems can accelerate initial implementation by mitigating contamination risks and easing regulatory approval processes, while gradually building local autonomy and expertise.

Human capital development will be a cornerstone of sustainability. Establishing compact, interdisciplinary teams with cross-training in GMP operations, quality assurance, clinical logistics, and data science is both feasible and cost-effective in LAC, given lower personnel costs relative to high-income countries. Additionally, embedding postgraduate education and professional training within GMP initiatives will strengthen the regional biotechnology workforce and foster long-term innovation capacity.

Looking ahead, the integration of artificial intelligence into GMP operations offers a realistic and scalable pathway for LAC. Early adoption of electronic batch records, soft sensors, and predictive analytics can improve process control and reduce variability, while staged implementation of digital twins and advanced model-predictive controls may transform local facilities into globally competitive hubs for CAR T-cell manufacturing. Equally, regional collaborations under the framework of PAHO’s Red PARF and related harmonization initiatives should be prioritized to ensure regulatory alignment, streamline approvals, and safeguard equitable access.

In conclusion, a phased, resource-adapted, and digitally enabled GMP laboratory model can bring CAR T-cell therapy closer to patients in LAC. The convergence of modular infrastructure, automated platforms, workforce development, and AI-driven process optimization provides a practical roadmap for democratizing advanced therapies in the region. Future research should focus on real-world implementation studies, cost-effectiveness analyses, and the development of governance frameworks that ensure quality, sustainability, and equitable patient access.

## Glossary

AI: Artificial Intelligence
ANMAT: Administración Nacional de Medicamentos, Alimentos y Tecnología Médica (Argentina)
ANVISA: Agência Nacional de Vigilância Sanitária (Brazil)
ASHRAE: American Society of Heating, Refrigerating and Air-Conditioning Engineers
CAR: Chimeric Antigen Receptor
CFU: Colony-Forming Unit
CMO: Contract Manufacturing Organization
COFEPRIS: Comisión Federal para la Protección contra Riesgos Sanitarios (Mexico)
CRT: Climate Room Test
DMSO: Dimethyl Sulfoxide
EBMT: European Society for Blood and Marrow Transplantation
eBR: Electronic Batch Record
EMA: European Medicines Agency
FACT: Foundation for the Accreditation of Cellular Therapy
FDA: Food and Drug Administration (United States)
GMP: Good Manufacturing Practice
HCT: Hematopoietic Cell Transplantation
HEPA: High Efficiency Particulate Air
HSCT: Hematopoietic Stem Cell Transplantation
HVAC: Heating, Ventilation, and Air Conditioning
INVIMA: Instituto Nacional de Vigilancia de Medicamentos y Alimentos (Colombia)
IQ: Installation Qualification
ISCT: International Society for Cell & Gene Therapy
ISO: International Organization for Standardization
ISPE: International Society for Pharmaceutical Engineering
JACIE: Joint Accreditation Committee ISCT–EBMT
LAC: Latin America and the Caribbean
LAL: Limulus Amebocyte Lysate
LN2: Liquid Nitrogen
MERV: Minimum Efficiency Reporting Value
NGS: Next-Generation Sequencing
OQ: Operational Qualification
PAHO: Pan American Health Organization
PBMC: Peripheral Blood Mononuclear Cells
PCR: Polymerase Chain Reaction
PPP: Public–Private Partnership
PQ: Performance Qualification
QA: Quality Assurance
QC: Quality Control
qPCR: Quantitative Polymerase Chain Reaction
RCL: Replication Competent Lentivirus
RH: Relative Humidity
SOP: Standard Operating Procedure
UPS: Uninterruptible Power Supply
WHO: World Health Organization
