# Supplementary material for: A proposal of good manufacturing practices (GMP) laboratory for CAR T-cell therapy in Latin America and the Caribbean: challenges and opportunities
Source: Front Public Health. 2026 Aug 20;14:1875553. doi: 10.3389/fpubh.2026.1875553 (PMC13539418; doi:10.3389/fpubh.2026.1875553)
Supplement: Supplementary file 1 [file Data_sheet_1.docx]

**Supplementary Table 1.** Price-per-infusion values used to generate the color scale in Figure 1. Together with the verification status and primary or secondary source for each country/region. Because most jurisdictions negotiate CAR T-cell prices confidentially (managed entry agreements, outcome-based payments, or lump-sum agreements), publicly disclosed net prices are unavailable for many countries; in these cases, the value shown reflects the best available public list price, a regional proxy, or an author estimate, and is labeled accordingly. Confidence tiers: High = value confirmed against an official/primary source (government price list, national HTA/reimbursement body, or peer-reviewed publication reporting the specific negotiated or list price). Moderate = value supported by a credible secondary or peer-reviewed source but not an official price list. Low = no independently verifiable public source was identified; the value is an author estimate and should be interpreted with caution or revised prior to publication.

| **Country / Region** | **Verified / recommended price (USD)** | **Confidence** | **Notes** |
| --- | --- | --- | --- |
| United States | ≈529,000 (2025 WAC average of 6 approved products) | High | Figure 2 uses 2017–2022 launch list prices. Current wholesale acquisition cost (WAC) is substantially higher (Kymriah $593,533; Yescarta $503,580; Tecartus $462,000; Breyanzi $531,350; Abecma $528,312; Carvykti $555,310) [91] . |
| Spain | ≈365,000–373,000 (official list price); ARI-0001 ≈103,000 (US$ equivalent of €89,270) | High | Official maximum price set by the Comisión Interministerial de Precios de los Medicamentos: €320,000 (Kymriah), €327,000 (Yescarta) [92] . In contrast, the official reimbursement price approved by the Spanish Ministry of Health for the academic CAR-T ARI-0001, developed at Hospital Clínic Barcelona, is approximately €89,270 [93] . |
| United Kingdom | ≈380,000 (list price, pre-confidential-discount) | High | NHS/NICE list price for Kymriah: £282,000; Yescarta list price ≈£300,000. Actual NHS net price is confidential and lower [92] . |
| Japan | ≈203,000 (current NHI price at 2026 JPY/USD rate) | High | National Health Insurance (NHI) price is JPY 32,647,761, identical for Kymriah, Yescarta, Breyanzi, Abecma, and Carvykti. Converted at the current exchange rate (~161 JPY/USD) [94] . |
| Singapore | ≈375,000 | Moderate | Reported list price for Kymriah/Yescarta in Singapore [94] . |
| South Korea | ≈300,000 | Moderate | Kymriah list price reported at approximately KRW 300,000,000; National Health Insurance Service (NHIS) covers the majority of the cost [94] . |
| China (commercial products) | ≈175,000 (commercial products, e.g., Fosun Kite/relmacabtagene autoleucel) | Moderate | Commercial, imported/licensed CAR-T products in China are reported near US$175,000; domestically developed academic/decentralized products are considerably cheaper (~US$40,000–80,000), consistent with the mixed values already used in the script [94] . |
| India | ≈36,000–37,000 (current), was ≈48,000 at launch | High | NexCAR19 (ImmunoACT) launch price was ₹30–42 lakh; current price has decreased to ≈₹30 lakh (≈US$36,000–37,000) as production has scaled [95] . |
| France / Germany / Italy / Netherlands / Belgium / Austria / Denmark / Finland / Luxembourg / Sweden / Portugal / Poland / Hungary / Czech Republic (EU list-price proxy) | ≈365,000 (EU5 reference price, €320,000 at current EUR/USD) | Low–Moderate | The figure 2 applies a distinct value per country without a documented country-specific source for most of these; only the EU5 reference price (based on the EMA-harmonized indication and the French/German list price of ~€320,000) is independently verifiable at the aggregate level. Country-specific negotiated (net) prices are confidential in essentially all EU member states (managed entry agreements), so per-country values below the EU5 reference should be treated as author estimates pending further sourcing [96] . |
| Switzerland | ≈375,000 | Moderate | List price reported at approximately CHF 300,000, converted at current CHF/USD rate. Actual reimbursed price is negotiated confidentially with Swiss authorities [92] . |
| Norway | ≈320,000 | Low–Moderate | List price for Kymriah reported at NOK 3,167,606, converted at current NOK/USD rate. Note that as of mid-2022, adult DLBCL patients in Norway reportedly could not access Kymriah/Yescarta within the public system, so this list price may not correspond to an active, funded pathway; recommend author verification of current status [92] . |
| Canada | 320,000 | Low | No official Canadian list or negotiated price is publicly disclosed (Canada uses confidential price negotiation via the pan-Canadian Pharmaceutical Alliance). The value in figure 2 is a plausible proxy but is not independently verifiable from a public source [92] . |
| Brazil | 150,000 — no direct public source located | Low | CAR-T is not reimbursed in Brazil's public health system; limited private-insurance availability exists. The $150,000 figure appears to reflect an estimated academic/point-of-care manufacturing cost rather than a commercial market price, and should be sourced explicitly (e.g., to a specific academic program) or clearly labeled as an author estimate [97] . |

**Supplementary Table 2.** ISO 14644-1 Air Classifications.

| **Clean area classification (0.5 µm/ft^3^ particles)** | **ISO designation** | **Particles >0.5 µm per m^3^** | **Microbiologically active air action levels (CFU/m^3^)** | **Microbiological action levels of the establishment plates (diam. 90 mm; CFU/4 hours)** |
| --- | --- | --- | --- | --- |
| 100 | 5 | 3,520 | 1 | 1 |
| 1,000 | 6 | 35,200 | 7 | 3 |
| 10,000 | 7 | 352,000 | 10 | 5 |
| 100,000 | 8 | 3,520,000 | 100 | 50 |

*CFU: colony-forming unit; ISO: international organization for standardization.*

**Supplementary Table 3.** Critical equipment for a GMP CAR T-cell therapy laboratory.

| **Equipment** | **General function** |
| --- | --- |
| Class II biosafety cabinets (A2 or B2) | Aseptic manipulations and operator/sample protection |
| CO₂ incubators (mammalian cell culture) | Controlled temperature/CO₂/humidity for cell expansion/incubation. |
| Magnetic cell separation systems (column-based and column-free) | Selection/depletion of subpopulations (CD4/CD8 or PBMC enrichment). |
| Closed/semi-automated expansion systems (single-use compatible) | Scalable culture with reduced contamination risk. |
| Dedicated viral transduction area/system | Vector containment and traceability |
| Centrifuges (benchtop/gradient) | Washes, concentration steps, and separations |
| Controlled-rate freezer + LN₂ storage tanks | Cryopreservation and final product storage |
| Shared in-house QC equipment   - qPCR - Flow cytometer - Endotoxin reader - Microbiology incubators | - Identity, transgene copy number - Immunophenotyping, viability - LAL - Sterility/mycoplasma per validated methods |

*LAL: limulus amebocyte lysate; LN₂: liquid nitrogen; PBMC: peripheral blood mononuclear cells; QC: quality control; qPCR: quantitative polymerase chain reaction.*

**Supplementary Figure 1.** Ideal requirements for establishing a GMP laboratory for CAR-T cell manufacturing

*DMSO: dimethyl sulfoxide; GMP: Good Manufacturing Practices; HEPA: High Efficiency Particulate Air; QC: quality control; qPCR: quantitative polymerase chain reaction.*

**Supplementary Figure 2.** Environmental controls in GMP laboratory.

**Supplementary Figure 3.** Quality control process in GMP laboratory for CAR T- cells.

*CAR T-cell: chimeric antigen receptor T-cell; DNA: deoxyribonucleic acid; GMP: good manufacturing practice; PCR: polymerase chain reaction; qPCR: quantitative polymerase chain reaction*

**Supplementary Figure 4.** Summary of FACT-JACIE International Standards for GMP (flowchart).

*GMP: good manufacturing practice; SOP: Standard Operating Procedures.*
